# Immune senescence and immune activation in elderly colorectal cancer patients

**DOI:** 10.18632/aging.102022

**Published:** 2019-06-13

**Authors:** Silvia Giunco, Maria Raffaella Petrara, Francesca Bergamo, Paola Del Bianco, Marisa Zanchetta, Francesco Carmona, Vittorina Zagonel, Anita De Rossi, Sara Lonardi

**Affiliations:** 1 Section of Oncology and Immunology, Department of Surgery, Oncology and Gastroenterology - DiSCOG, University of Padova, Padova 35128, Italy; 2 Medical Oncology Unit 1, Veneto Institute of Oncology IOV – IRCCS, Padova 35128, Italy; 3 Clinical trials and Biostatistic Unit, Veneto Institute of Oncology IOV – IRCCS, Padova 35128, Italy; 4 Immunology and Molecular Oncology Unit, Veneto Institute of Oncology IOV – IRCCS, Padova 35128, Italy

**Keywords:** immune senescence, inflammation/immune activation, colorectal cancer, elderly

## Abstract

In our previous study, we found that low thymic output and short telomere length were associated with a higher risk of tumor in elderly cancer patients. Here, we aimed to examine in depth the impact of immunological and biological senescence and immune activation on disease outcome in elderly patients with colorectal cancer (CRC).Peripheral blood samples from 81 CRC patients were studied for immune activation, immune senescence and recent thymic emigrant(RTE) CD4 and CD8 cells by flow cytometry. T-cell receptor rearrangement excision circle (TREC) levels and telomere lengths were measured by real-time PCR. Plasma levels of microbial translocation markers, LPS and sCD14, were quantified by ELISA. While TREC levels and telomere length were not prognostic of disease outcome, high percentages of immune senescent and immune activated CD8 cells were associated with a higher risk of a negative event (relapse, progression, or death) in all studied patients and disease relapse in I-III staged patients. Levels of sCD14 and LPS were higher in patients who will experience a negative event than in patients who will not. In conclusion, in elderly CRC patients higher immunological senescence and immune activation negatively impact the disease outcome; how these characteristics influence the antineoplastic treatments remains to be investigated.

## Introduction

In 2018, there were an estimated 140,250 new cases of colorectal cancer (CRC), making it the third most common type of cancer in both men and women [1]. CRC was also responsible for over 50,000 deaths, leading to an estimated 5-year overall survival (OS) of 64.5% [1].

Age is considered a major risk for solid cancers [2], since cell senescence is associated with oncogene activation, DNA damage accumulation, mitochondrial dysfunction [3] and low-grade chronic inflammation, responsible for age-related diseases [4]. Immune senescence refers to the continuous remodeling of lymphoid organs, leading to reduced immune functions in elderly populations [5].

In clinical practice, increasing numbers of elderly patients with CRC undergo surgery and/or receive chemotherapy. These individuals are more likely than young patients to have comorbidities, such as cardiovascular disease, respiratory disease, renal dysfunction, and/or liver dysfunction, lowering the benefit/risk balance compared to younger people [6].

In our first exploratory study, immune senescence was significantly higher in elderly cancer patients than in age-matched controls; in particular, cancer patients, compared to controls, exhibited similar percentages of senescent CD4 lymphocytes, but higher percentages of senescent CD8 cells [7]. In agreement with this observation, we found that elderly cancer patients have significantly lower thymic output and shorter telomeres in their peripheral blood cells than age-matched non-cancer patients. Lower thymic output and shorter telomeres were associated with a higher risk of developing cancer [7].

The development of CRC from normal colonic epithelia requires a series of genetic and inflammatory-immunological factors to enable and shape a tumorigenic milieu [8]. Studies on the implications of the gut microbiota in CRC are now increasing; activation of immune signaling pathways by bacterial stimuli may result in the loss of homeostasis that drives a pro-neoplastic inflammatory environment [8]. Indeed, damage to intestinal mucosa may promote translocation of microbial products, defined “pathogen-associated molecular patterns” (PAMPs), into circulation. PAMPs, such as lipopolysaccharide (LPS) and soluble (s)CD14, can activate the immune system through Toll-like receptors (TLRs) and NF-κB pathway, and this activation mediates the production of several pro-inflammatory cytokines and chemokines, such as interleukin (IL)-6, tumor necrosis factor (TNF) and cyclooxygenase 2, which are associated with tumor development and progression [8–10].

To date, while there are few data concerning the impact of immune senescence, no data are available about the role of immune activation on disease outcome in elderly cancer patients. In the present study, we explored the prognostic role of immune senescence and immune activation in a cohort of elderly CRC patients.

## RESULTS

### Characteristics of the study population

Eighty-one patients with colorectal cancer were enrolled in this study. Their clinical and biological characteristics at baseline are reported in Table 1. Median age was 76 years; 60.5% were male. According to TNM staging system, 3 patients (3.7%) had stage I, 22 (27.2%) stage II, 36 (44.4%) stage III and 20 (24.7%) stage IV. A comprehensive geriatric assessment (CGA) was performed in all patients; 55 were classified as fit (67.9%), 20 were vulnerable (24.7%) and 6 were frail (7.4%). Seventy patients (86.4%) received a systemic treatment in an adjuvant (63.0%) and/or metastatic setting (37.0%), while 11 patients (13.6%) were not treated, due to frailty recognized at CGA or early stage of disease with no indication to adjuvant treatment.

**Table 1:** Clinical and biological characteristics of CRC patients.

		**All patients** **(n. 81)**	**Patients on** **stage I-III** **(n. 61)**	**Patients on** **stage IV** **(n. 20)**
**Age (years)**	Mean (SD)	77.1 (4.1)	77.1 (3.8)	76.9 (5.2)
	Median (IQR)	76 (74-80)	76 (75-80)	78 (71.5-81.5)
**Gender**	F	32 (39.5%)	27 (44.3%)	5 (25.0%)
	M	49 (60.5%)	34 (55.7%)	15 (75.0%)
**Geriatric assesment**	Fit	55 (67.9%)	40 (65.6%)	15 (75.0%)
	Vulnerable	20 (24.7%)	17 (27.9%)	3 (15.0%)
	Frail	6 (7.4%)	4 (6.6%)	2 (10.0%)
**Stage**	I	3 (3.7%)	3 (4.9%)	
	II	22 (27.2%)	22 (36.1%)	
	III	36 (44.4%)	36 (59.0%)	
	IV	20 (24.7%)		20 (100%)
**Site**	Right	34 (42.0%)	29 (47.5%)	5 (25.0%)
	Left	34 (42.0%)	25 (41.0%)	9 (45.0%)
	Rectum	13 (16.0%)	7 (11.5%)	6 (30.0%)
**Adjuvant therapy**	No	30 (37.0%)	14 (22.9%)	16 (80.0%)
	Yes	51 (63.0%)	47 (77.1%)	4 (20.0%)
**IL6**	Mean (SD)	4.0 (2.9)	3.2 (2.0)	6.2 (4.0)
	Median (IQR)	2.8 (1.9-4.5)	2.6 (1.9-3.9)	4.8 (3.5-9.0)
**CRP**	Mean (SD)	12.2 (24.6)	8.1 (22.1)	25.0 (28.2)
	Median (IQR)	3.3 (2.9-8.9)	2.9 (2.9-4.5)	12.6 (4.9-34.8)
**Telomere length (TS)**	Mean (SD)	1.3 (0.3)	1.3 (0.3)	1.4 (0.4)
	Median (IQR)	1.4 (1.1-1.5)	1.3 (1.1-1.5)	1.5 (1.0-1.7)
**TREC/10^5^ PBMC**	Mean (SD)	48.6 (58.0)	46.1 (56.9)	56.1 (62.2)
	Median (IQR)	26.5 (10.5-62.0)	22.5 (11.0-58.0)	42.0 (6.0-64.0)
**% CD8 recent thymic emigrant cells**	Mean (SD)	38.0 (17.3)	38.4 (18.6)	36.6 (13.1)
**(CD8+CD45RA+CD31+)**	Median (IQR)	37.7 (25.0-49.2)	37.4 (26.2-51.1)	37.7 (23.8-42.3)
**% CD4 recent thymic emigrant cells**	Mean (SD)	18.1 (11.8)	19.3 (12.5)	14.8 (9.2)
**(CD4+CD45RA+CD31+)**	Median (IQR)	15.8 (9.0-24.1)	16.8 (9.0-27.9)	13.4 (9.1-19.0)
**% CD8 activated cells**	Mean (SD)	5.4 (6.1)	4.7 (6.1)	7.6 (6.0)
**(CD8+CD38+HLA-DR+)**	Median (IQR)	3.2 (2.2-5.2)	2.7 (2.0-4.3)	5.2 (3.1-9.8)
**%CD8 senescent cells**	Mean (SD)	44.7 (15.7)	42.4 (14.8)	49.8 (16.8)
**(CD8+CD28-CD57+)**	Median (IQR)	44.0 (34.6-53.7)	41.2 (32.7-51.5)	47.4 (39.6-61.8)
**%CD4 senescent cells**	Mean (SD)	9.9 (12.5)	10.6 (13.4)	8.5 (10.8)
**(CD4+CD28-CD57+)**	Median (IQR)	5.1 (1.9-12.7)	5.3 (2.4-12.6)	4.8 (1.2-13.0)
**CD4/CD8 ratio**	Mean (SD)	2.3 (1.7)	2.3 (1.7)	2.4 (1.7)
	Median (IQR)	1.8 (1.2-3.0)	1.7 (1.1-3.0)	2.1 (1.3-2.8)

### Immune senescence markers

**Figure 1:**
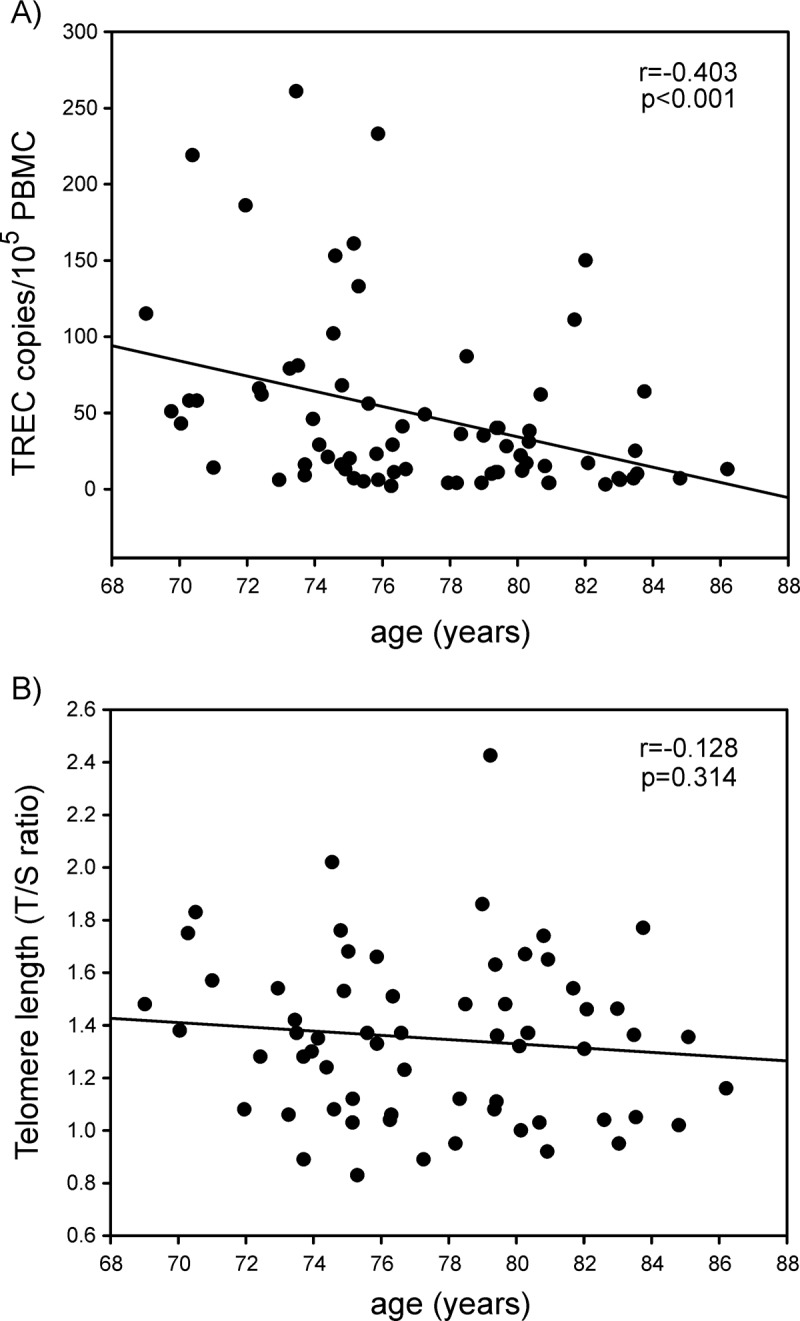
**Correlation between age and (A) TREC levels, and (B) telomere length in cancer patients.** (**A**) TREC levels decline with age in peripheral blood cells of cancer patients; (**B**) Relative telomere length (T/S) did not decrease with age in peripheral blood cells of cancer patients.

TREC levels decreased significantly with increasing age in patients (r_s_=−0.403; p<0.001) (Figure 1A), while telomere length did not correlate with age (r_s_=−0.128, p=0.314) (Figure 1B). These findings are in agreement with our first exploratory study [7]. Consistently with the knowledge that TREC is a marker for thymic output, a positive correlation was found between TREC and CD4 recent thymic emigrant (RTE) cells (r_s_=0.270, p=0.026), and CD8 RTE cells (r_s_=0.290, p=0.022); of interest, there was a trend to negative correlation between TREC and activated CD8 cells (r_s_=-0.230, p=0.054). Levels of TREC and length of telomeres were not prognostic of disease relapse in the disease-free-survival (DFS) conducted in the 61 I-III staged patients (Table 2), neither of a negative outcome event (relapse/progression or death) in the event-free-survival (EFS) performed in all 81 patients included in the study (Table 3).

**Table 2:** Univariate Cox regression models of disease-free-survival (DFS) in 61 CRC patients on stage I-III.

		** no. event/total number**	**hazard ratio**	**95%CI**	***p-value***
**Age (years)**	continuous		1.0	0.8-1.1	*0.6965*
**Gender**	F	8/27	1		
	M	7/34	0.8	0.3-2.2	*0.6948*
**Stage**	I-II	5/25	1		
	III	10/36	1.6	0.6-4.8	*0.3671*
**Site**	Right	8/29	1		
	Left	6/25	0.9	0.3-2.5	*0.7845*
	Rectum	1/7	0.5	0.1-4.1	*0.5218*
**Adjuvant therapy**	No	5/14	1.8	0.6-5.3	*0.2833*
	Yes	10/47	1		
**Geriatric assessment**	Fit	11/40	1		
	Vuln/Frail	4/21	0.7	0.2-2.2	*0.4911*
**IL6**	<=5.31	12/54	1		
	>best	2/4	5.6	1.1-28.7	***0.0368***
**CRP**	<=5.94	11/49	1		
	>best	4/10	2.2	0.7-7.0	*0.1682*
**Telomere length (T/S)**	<=1.37	7/33	1		
	>best	6/16	1.9	0.6-5.6	*0.2644*
**TREC/10^5^ PBMC**	<=40	9/37	1		
	>best	6/17	1.6	0.6-4.5	*0.3726*
**% CD8 recent thymic emigrant cells**	<=33.53	1/23	1		
**(CD8+CD45RA+CD31+)**	>best	12/33	8.4	1.1-64.7	***0.0410***
**% CD4 recent thymic emigrant cells**	<=21.42	10/35	1.8	0.6-5.8	*0.3065*
**(CD4+CD45RA+CD31+)**	>best	4/21	1		
**% CD8 activated cells**	<=6.7	11/53	1		
**(CD8+CD38+HLA-DR+)**	>best	3/5	4.1	1.1-15.0	***0.0297***
**%CD8 senescent cells**	<=43.96	4/25	1		
**(CD8+CD28-CD57+)**	>best	8/17	3.5	1.0-11.6	***0.0434***
**%CD4 senescent cells**	<=12.83	6/32	1		
**(CD4+CD28-CD57+)**	>best	4/9	2.4	0.7-8.6	*0.1680*
**CD4/CD8 ratio**	<=0.68	4/6	7.0	2.1-23.3	***0.0014***
	>best	10/54	1		

**Table 3:** Univariate Cox regression models of event-free-survival (EFS) in 81 CRC patients.

				**no. event/** **total number**	**hazard ratio**		**95%CI**	***p-value***
**Age (years)**		continuous			1.0		0.9-1.1	*0.7154*
**Gender**		F		13/32	1			
		M		22/49	1.4		0.7-2.7	*0.3839*
**Stage**		I-III		15/61	1			
		IV		20/20	35.4		12.3-101.1	***<0.0001***
**Site**		Right		13/34	1			
		Left		15/34	1.2		0.6-2.5	*0.6199*
		Rectum		7/13	1.9		0.8-4.8	*0.1684*
**Adjuvant therapy**		No		21/30	3.8		1.9-7.5	***0.0001***
		Yes		14/51	1			
**Geriatric assessment**		Fit		26/55	1			
		Vulnerable		6/20	0.5		0.2-1.3	*0.1772*
		Frail		3/6	1.1		0.3-3.7	*0.8616*
**IL6**		<=5.31	23/65		1			
		>best	11/13		5.4	2.5-11.8		***<0.0001***
**CRP**		<=5.94	16/54		1			
		>best	18/24		4.6	2.3-9.1		***<0.0001***
**Telomere length (T/S)**		<=1.38	14/40		1			
		>best	14/24		1.9	0.9-4.1		*0.0783*
**TREC/10^5^ PBMC**		<=40	17/45		1			
		>best	16/27		1.9	0.9-3.7		*0.0700*
**% CD8 recent thymic emigrant cells**		<=33.53	7/29		1			
**(CD8+CD45RA+CD31+)**		>best	25/46		2.4	1.1-5.7		***0.0369***
**% CD4 recent thymic emigrant cells**		<=21.42	28/53		2.8	1.2-6.9		***0.0203***
**(CD4+CD45RA+CD31+)**		>best	6/23		1			
**% CD8 activated cells**		<=6.7	22/64		1			
**(CD8+CD38+HLA-DR+)**		>best	12/14		4.4	2.1-9.1		***<0.0001***
**%CD8 senescent cells**		<=43.96	10/31		1			
**(CD8+CD28-CD57+)**		>best	21/30		3.0	1.4-6.3		***0.0051***
**%CD4 senescent cells**		<=12.83	20/46		1			
**(CD4+CD28-CD57+)**		>best	9/14		1.6	0.7-3.5		*0.2245*
**CD4/CD8 ratio**		<=0.68	5/7		2.5	1.0-6.5		*0.0615*
		>best	28/72		1			

**Figure 2:**
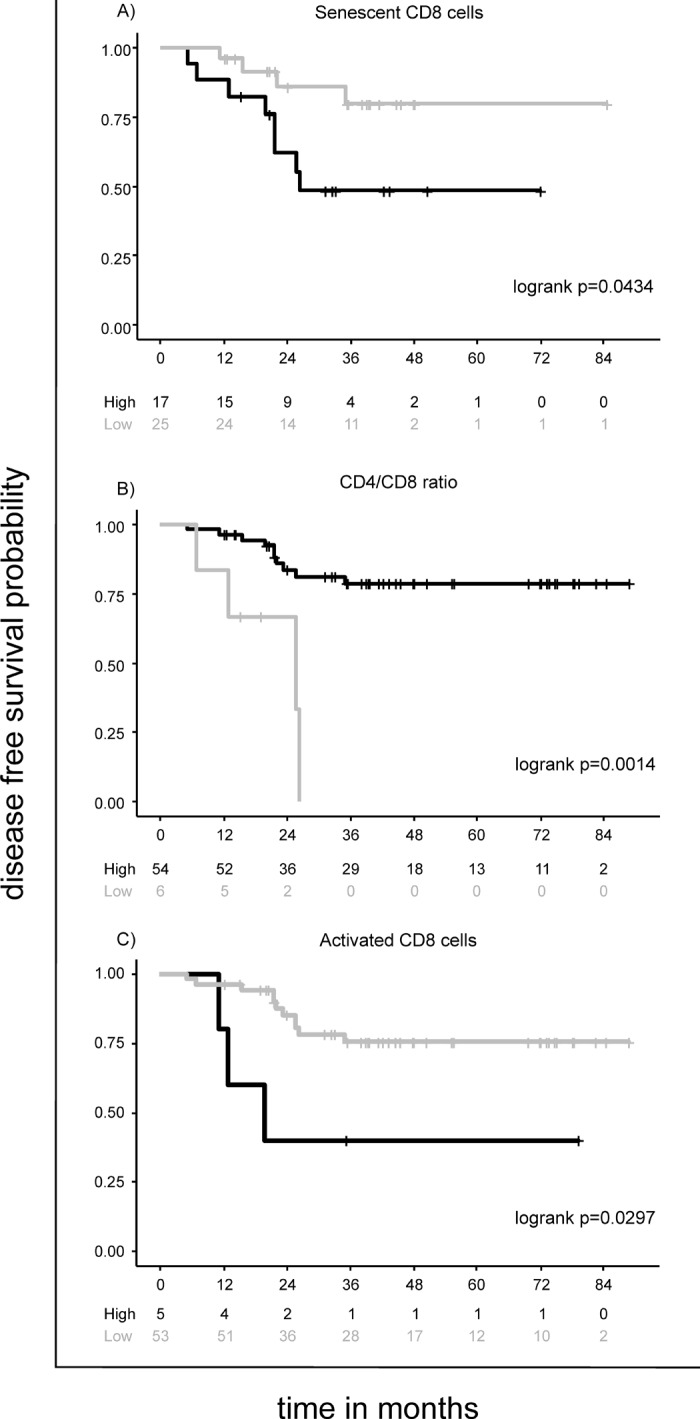
**Disease-free-survival according to levels of immune senescence and immune activation.** Kaplan–Meier curves for disease-free survival (DFS) analyses in the 61 I-III staged patients according to best cut-off levels of (**A**) senescent CD8 cells, (**B**) CD4/CD8 ratio, (**C**) activated CD8 cells.

Among the immunological cellular subtypes, high percentage (up to the best cut-off value) of senescent CD8 cells, but not CD4 cells, was significantly associated with a higher risk of disease relapse in the DFS (Hazard Ratio (HR) [95% Confidence Interval]= 3.5 [1.0-11.6], p=0.0434) (Table 2 and Figure 2A), and higher risk of a negative event in the EFS (HR=3.0 [1.4-6.3], p=0.0051) (Table 3). Notably, senescent CD8 cells were highly expressed in IV staged patients (Table 1) and significantly associated with disease stage (I-III *vs* IV p=0.0043); when adjusted for stage (I-III *vs* IV), this marker missed the association with EFS (HR adjusted =1.8 [0.8-4.0], p=0.1470) (not shown).

Low percentage of CD4 RTE cells was not prognostic of DFS (Table 2). Its prognostic value of EFS (HR=2.8 [1.2-6.9], p=0.0203) (Table 3) was lost when adjusted for stage (HR adjusted=1.6 [0.6-4.1], p=0.2956). Interestingly, high percentages of CD8 RTE cells were prognostic of both DFS (HR= 8.4 [1.1-64.7], p=0.0410) (Table 2) and EFS (HR= 2.4 [1.1-5.7], p=0.0369) (Table 3), and remained independently associated to EFS, after adjusting for stage (HR adjusted= 2.4 [1.0-5.7], p=0.0442].

The CD4/CD8 ratio was investigated as a surrogate marker of immune senescence [11]. While low CD4/CD8 ratio (<0.68) lacked of prognostic role in the EFS (Table 3), I-III staged patients with low CD4/CD8 ratio were at higher risk of disease relapse (HR= 7.0 [2.1-23.3], p=0.0014) (Table 2 and Figure 2B).

### Immune activation markers

High percentages (up to the best cut-off value) of immune activated CD8 cells were significantly associated with higher risk of disease relapse in the DFS (HR= 4.1 [1.1-15.0], p=0.0297) (Table 2 and Figure 2C), and negative event in the EFS (HR= 4.4 [2.1-9.1]; p<0.0001) (Table 3). As pointed out for senescent CD8 cells, the activated CD8 cells were highly expressed in IV staged patients and significantly associated to disease stage (I-III *vs* IV p=0.0003); when adjusted for stage (I-III *vs* IV), this marker missed the association with EFS (HR adjusted =1.2 [0.5-2.7], p=0.7218) (not shown).

CRC patients with high levels of circulating pro-inflammatory cytokines IL-6 and C reactive protein (CRP) showed higher risk of disease relapse in the DFS (HR= 5.6 [1.1-28.7], p=0.0368 for IL-6 and 2.2 [0.7-7.0], p=0.1682 for CRP, Table 2) and negative event in the EFS (HR= 5.4 [2.5-11.8], p<0.0001 for IL-6 and 4.6 [2.3-9.1], p<0.0001 for CRP, Table 3).

**Figure 3:**
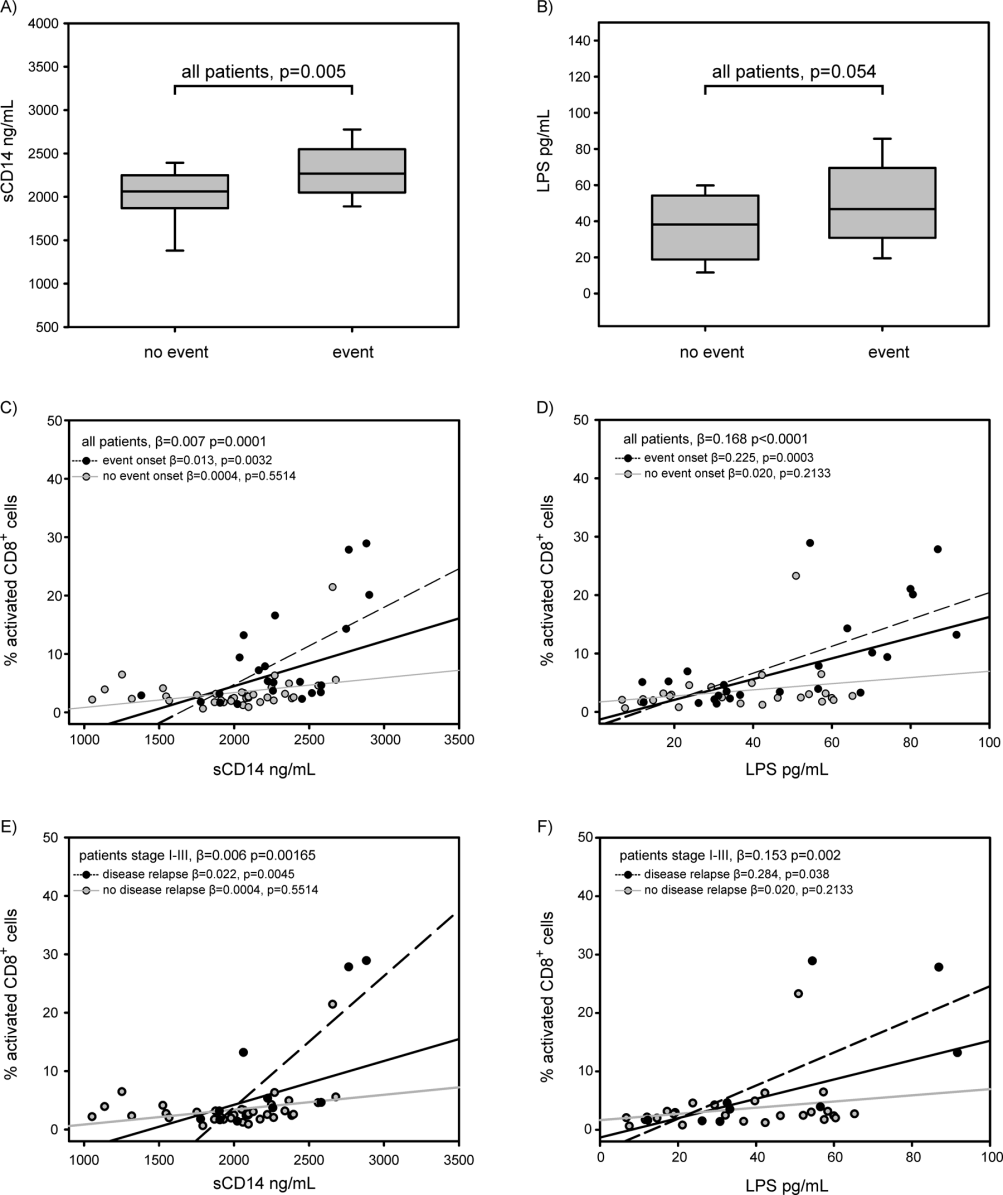
**PAMPs and immune activation.** (**A**) sCD14 and (**B**) LPS levels in patients who will or not experience a negative event (relapse or progression or death). (**C**) sCD14 and (**D**) LPS levels as function of percentages of activated CD8^+^ cells in all patients. Regression line for all 81 patients is represents in bold black; patients who will experience a negative event are represented as black circles and medium dash line, and patients who will not as grey circles and line. (**E**) sCD14 and (**F**) LPS levels as function of percentages of activated CD8 cells in I-III staged patients. Regression line for all 61 patients is represent in bold black; patients who will have a disease relapse are represented as black circles and medium dash line, and patients who will not as grey circles and line.

Median [interquartile-IQR] levels of sCD14 and LPS were higher in patients who will experience a negative event than in patients who will not (2268 [2050-2549] *vs* 2062 [1870-2249] ng/ml; p=0.005, for sCD14; 47 [31-70] *vs* 38 [19-54] pg/ml; p=0.054, for LPS) (Figure 3A and 3B). In I-III staged patients, levels of sCD14 tended to be higher in patients who will have a disease relapse than in those who will not (2224 [1906-2581] *vs* 2062 [1850-2253] ng/ml; p=0.063), while no differences were found for LPS levels (45 [31-87] *vs* 38 [18–55] pg/ml; p=0.189) (not shown).

Circulating levels of sCD14 and LPS were significantly increased with increasing percentages of activated CD8 cells in all patients (regression coefficient β=0.007, p=0.0001 for sCD14, and β=0.168, p<0.0001 for LPS) (Figure 3C and D). Notably, both sCD14 and LPS were strongly associated with activated CD8 in the group of patients who will experience a negative event (β= 0.013, p=0.0032 for sCD14, and β= 0.225, p=0.0003 for LPS), but not in the group of patients who will not (β=0.0004, p=0.5514 for sCD14, and β=0.020, p=0.2133 for LPS) (Figure 3C and 3D). Moreover, also in 61 I-III staged patients, levels of sCD14 and LPS were significantly increased with increasing immune activation. In particular, circulating levels of sCD14 and LPS were associated with increased percentages of activated CD8 in patients who will have a disease relapse (β= 0.022, p=0.0045 for sCD14, and β= 0.284, p=0.038 for LPS). (Figure 3E and 3F).

## DISCUSSION

This prospective study was planned as a logical continuation of our first exploratory study on immune senescence and onset of cancer in elderly patients [7] to explore in depth the impact of immune senescence and immune activation on the disease outcome (relapse/progression or death). To our knowledge, this is the first study that addresses both these specific aspects in elderly cancer patients.

In the previous study, we found that low TREC levels and low telomere length, markers of low thymic function and replicative senescence respectively, are associated with a higher risk of tumor onset [7]. In the present population of cancer patients, in agreement with the previous study, we found that TREC levels decline with age, while telomere length does not. However, both these parameters lack of prognostic role for disease outcome in elderly cancer patients.

Nonetheless, the immunological profile of T cells presents interesting aspects. Senescence might have tumor promoting effects, since senescent cells are avid secretors of pro-inflammatory cytokines and growth factors, known as the senescence associated secretory phenotype (SASP) and this secretion has been implicated in both aging and cancer development [12]. Interestingly, we found that senescent CD8 cells, but not CD4, displayed a significant relationship with disease outcome. Patients with high percentage of CD8 senescent cells had a worse disease outcome in both EFS and DFS analyses. The finding that only CD8 compartment is associated with a worse disease outcome may be consistent with the concept that CD8 T cells are the key components of tumor immune surveillance, and, thus, the tumor-induced dysfunction may be more evident in the CD8-cell subset than in the CD4-cell compartment [7,13,14].

Gut microbiome and inflammation are hypothesized to shape the tumour microenvironment and promote tumorigenesis [15–18]. A relationship between commensal bacteria and intestinal carcinogenesis was first suggested in rodent models by Reddy et al. in 1974 [19]. In recent studies, detailed microbial profiles gained by high-throughput analyses, like metagenomics sequencing and 16S rDNA sequencing, have revealed that bacteria are associated with CRC [20–23]. However, nothing was reported to our knowledge about cellular immune activation. We found that high percentages of CD8 activated cells were significantly associated with worse disease outcome. Moreover, circulating levels of microbial translocation markers (sCD14 and LPS), i.e. PAMPs released from damaged intestinal mucosa, were correlated with higher percentages of CD8 activated cells in patients who relapse/progress to disease. This association strengthens the concept that microbial PAMPs, released from intestinal mucosa, activate the immune system against non-tumor antigen and promote escape (immune-evasion) and expansion of tumor cells, thus negatively influencing the disease outcome.

CD4/CD8 ratio has been used as a surrogate marker of immune senescence in the general population [11]. Several studies have identified that CD4/CD8 ratio decrease over the lifespan, and inversion of the CD4/CD8 ratio in the elderly has been associated with risk of frailty and chronic viral infections, such as cytomegalovirus [24,25]. In this study, we first described that a low CD4/CD8 ratio is a prognostic marker of disease relapse in I-III staged patients. Interestingly, also high percentages of CD8 RTE lymphocytes were associated with a poor clinical outcome in these patients. It has been reported that RTE cells, recently emigrated from the thymus, are lower in cancer patients than in controls [7]. We can speculate that the high levels of senescent and activated CD8 cells in patients with progression of disease may induce a recall of new CD8 cells from the thymus. Increased levels of senescent, activated and RTE CD8 cells contribute to the low CD4/CD8 ratio.

In conclusion, in elderly colorectal cancer patients, both the senescent phenotype and the damage of gut mucosa, with release of microbial products, induce immune activation and pro-inflammatory status that negatively impact the disease outcome. How these features might influence the benefit of anti-tumoral therapies in elderly cancer patients should be addressed in further studies.

## MATERIALS AND METHODS

### Study population

A total of 81 patients aged ≥70 years with new diagnosis of stage I-IV colorectal cancer, consecutively admitted to Unit of Medical Oncology 1, Istituto Oncologico Veneto-IRCCS, between October 2010 and May 2017, were enrolled into the study. Clinical, biomolecular and pathological features for each patient were collected in a specific database. Treatments for limited and advanced disease were collected. Each patient performed a comprehensive geriatric assessment (CGA) and a CT scan at baseline to define the stage of the disease and subsequently as for clinical practice. A blood sample was collected at tumor diagnosis for each patient. Data about white blood cells count, IL-6 and C-Reactive Protein (CRP) were registered.

The present study was approved by the Institutional Ethics Committee (CE IOV 2010/18) and conducted according to the Declaration of Helsinki and the guidelines of Good Clinical Practice. Written informed consent was obtained from all patients.

### Biomarkers analyses

### *Sample collection*

Peripheral blood mononuclear cells (PBMC) were isolated from peripheral blood by centrifugation on a Ficoll-Paque (Pharmacia, Uppsala, Sweden) gradient. PBMC and plasma samples were cryopreserved and stored, respectively, in liquid nitrogen and at -80°C until they were employed.

### *Flow cytometric analysis*

Approximately 250,000 PBMC were stained for 15 min in the dark using the Live/Dead Fixable Near-IR Dead Cell Stain Kit (Life Technologies, Carlsbad, CA, USA) and the following labelled monoclonal antibodies (mAbs): anti-CD3 [fluorescein isothiocyanate (FITC)], anti-CD4 [peridinin chlorophyll protein (PerCP)], anti-CD8 (PerCP), anti-CD31 [phycoerythrin (PE)], anti-CD45RA [allophycocyanin (APC)], anti-CD38 PE, anti-HLA-DR APC, anti-CD57 PE and anti-CD28 APC. All samples were analysed by four-color flow cytometer FACSCalibur (Becton Dickinson) equipped with a 488 nm argon-ion laser and a 635 nm red diode laser. A total of 50,000 events was collected in the lymphocyte gate using morphological parameters (forward- and side-scatter). Data were processed using CellQuest Pro Software (Becton Dickinson) and analysed using Kaluza^®^ Analysis Software v.1.2 (Beckman Coulter, Inc, Fullerton, CA, USA). The gating strategy for flow cytometry analysis to identify CD4+ and CD8+ cell subsets is described in Supplementary Figure 1.

### *Quantification of T-cell receptor rearrangement excision circle (TREC)*

Thymic output in PBMC was studied by measuring TREC levels by real-time polymerase chain reaction (PCR), as previously described [26]. TREC levels were expressed as TREC copy number/10^5^ PBMC [26,27].

### *Determination of telomere length*

Relative telomere length (T/S) was determined by monochrome quantitative multiplex PCR assay as previously described [28].

### Quantification of microbial translocation markers 

Plasma levels of lipopolysaccharide (LPS) levels were quantified with the Human Lipopolysaccharides ELISA Kit (Cusabio, Wuhan, China), and results were expressed as LPS ng/ml plasma. Plasma levels of human sCD14 were determined with the Quantikine Human sCD14 Immunoassay (R&D Systems Inc. Minneapolis, MN, USA), according to manufacturer’s instructions, and results were expressed as sCD14 ng/ml plasma.

### Statistical analysis

Disease-free survival (DFS) was evaluated for the 61 I-III staged patients as the time from sample collection to disease relapse. Event free survival (EFS) was evaluated for all patients as the time from sample collection to a negative event defined as relapse, progression, or death. Patients who did not develop any event during the study period were censored at the date of last observation.

The association of clinical characteristics and biological variables with survival was investigated through univariate Cox proportional hazards regression model, after checking any deviation from the proportional hazards assumption. In order to identify low and high risk group of patients, the immune senescence and immune activation variables were dichotomized with cut points corresponding to the most significant relation with the outcome, estimated from maximally selected log-rank statistic for values between the 10% and 90% quantile using the upper bound of the p-value by Hothorn and Lausen [29]. Hazard ratios were provided with their 95% confidence interval and were also adjusted for statistically significant clinical factors. Median EFS and DFS were estimated using the Kaplan-Meier method.

Patients were allocated in two groups according to the presence or not of any outcome event, and differences in microbial translocation markers variables between the groups were investigated by Wilcoxon rank-sum test. Linear correlation with age was assessed using the Spearman’s rank correlation coefficient. The relationship between activated CD8+ cells and sCD14 or LPS was fitted using linear regression models.

All statistical tests used a two-sided 5% significance level. Statistical analyses were performed using the SAS statistical package (SAS, rel. 9.4; SAS Institute Inc.), RStudio (RStudio: Integrated Development for R. RStudio, Inc., Boston, MA), and the Maxstat packages of R software (www.r-project.org/).

## Supplementary Material

Supplementary Figure
