# Supplementary material for: Immune senescence and immune activation in elderly colorectal cancer patients
Source: Aging (Albany NY). 2019 Jun 13;11(11):3864–75. doi: 10.18632/aging.102022 (PMC6594805; doi:10.18632/aging.102022)
Supplement: Supplementary Figure [file aging-11-102022-s001.pdf]

## SUPPLEMENTARY FIGURE

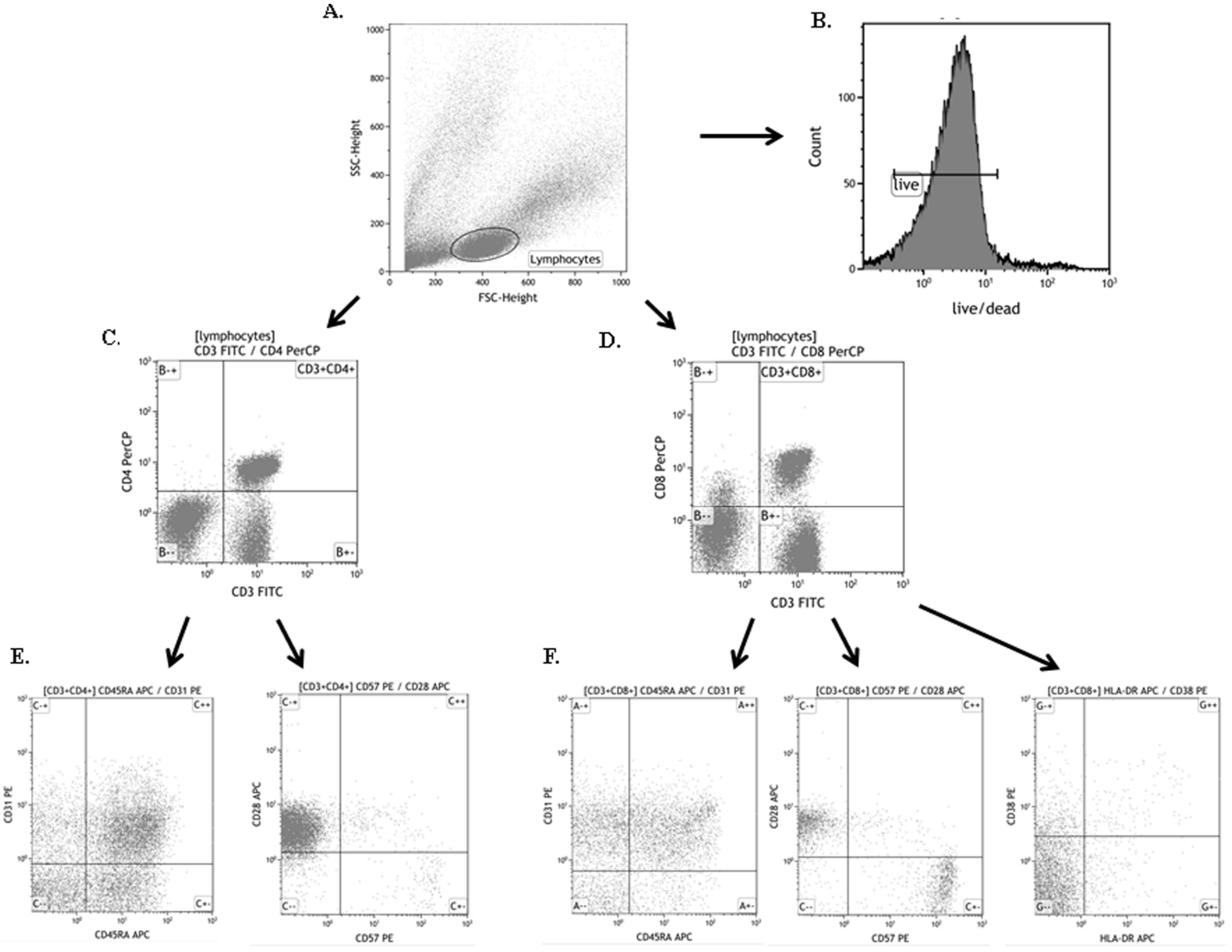


**Supplementary Figure 1. Gating strategy for flow cytometry analysis to identify CD4$^+$ and CD8$^+$ cell subsets.** (**A**) Cells were first gated for lymphocytes (FSC *versus* SSC). (**B**) Lymphocyte gate was in parallel analysed for uptake of the Live/Dead Fixable Near-IR Dead to determine live *versus* dead cells. The expression of (**C**) CD3$^+$CD4$^+$ or (**D**) CD3$^+$CD8$^+$ was determined on morphological gate for lymphocytes. (**E**) Within CD3$^+$CD4$^+$, CD45RA and CD31 markers were used to identify recent thymic emigrant (RTE, CD45RA$^+$CD31$^+$) and CD57 and CD28 markers for senescent cells (CD28$^-$CD57$^+$). (**F**) Within CD3$^+$CD8$^+$ gate, CD45RA and CD31 markers were used to identify recent thymic emigrant (RTE, CD45RA$^+$CD31$^+$), CD57 and CD28 markers for senescent cells (CD28$^-$CD57$^+$), and CD38 and HLA-DR for activated cells (CD38$^+$HLA-DR$^+$).
